# Association between the Microsatellite Ap243, AC117 and SV185 Polymorphisms and *Nosema* Disease in the Dark Forest Bee *Apis mellifera mellifera*

**DOI:** 10.3390/vetsci8010002

**Published:** 2020-12-29

**Authors:** Nadezhda V. Ostroverkhova

**Affiliations:** 1 Invertebrate Zoology Department, Biology Institute, National Research Tomsk State University, 36 Lenina Avenue, 634050 Tomsk, Russia; nvostrov@mail.ru; Tel.: +7-3822-529-461; 2 Department of Biology and Genetics, Siberian State Medical University, 2 Moskovsky Trakt, 634055 Tomsk, Russia

**Keywords:** *Nosema* disease, dark forest bee, *Apis mellifera mellifera*, microsatellite loci, association

## Abstract

The microsporidian *Nosema* parasites, primarily *Nosema ceranae*, remain critical threats to the health of the honey bee *Apis mellifera*. One promising intervention approach is the breeding of *Nosema*-resistant honey bee colonies using molecular technologies, for example marker-assisted selection (MAS). For this, specific genetic markers used in bee selection should be developed. The objective of the paper is to search for associations between some microsatellite markers and *Nosema* disease in a dark forest bee *Apis mellifera mellifera*. For the dark forest bee, the most promising molecular genetic markers for determining resistance to nosemosis are microsatellite loci AC117, Ap243 and SV185, the alleles of which (“177”, “263” and “269”, respectively) were associated with a low level of *Nosema* infection. This article is the first associative study aimed at finding DNA loci of resistance to nosemosis in the dark forest bee. Nevertheless, microsatellite markers identified can be used to predict the risk of developing the *Nosema* disease.

## 1. Introduction

In the last few decades, negative processes such as massive losses of bee colonies and hybridization have been observed in honey bee populations worldwide. The honey bee colony losses, called colony collapse disorder (CCD), as a result of reduced adaptation of honey bees to environmental factors, pose a threat to beekeeping worldwide [1,2]. It has been suggested that CCD can be caused by many causes, including various diseases such as nosemosis, environmental pollution, exposure to pesticides, weather and agricultural and beekeeping practices [3,4,5]. On the one hand, in order to avoid a catastrophic population decline from pests and diseases, it is necessary to maintain a high level of genetic diversity in honey bee populations [6,7]. On the other hand, molecular technologies, for example marker-assisted selection (MAS) can be used to identify bee colonies carrying specific traits of interest (e.g., resistance to pathogens and parasites, gentleness and high honey productivity) or the lack of undesirable traits (e.g., aggression and swarming) [8,9,10,11]. Marker-assisted selection is a new technology in beekeeping, and no specific genetic markers that could be used in bee breeding have been proposed [12,13].

The search for informative DNA loci/genes associated with economically useful and other traits (associative mapping) is highly relevant. The preferred strategy is to genotype a high number of genetic markers in linkage or association studies in order to identify genomic regions and discover the causative genes [14]. The identification of genetic markers associated with the phenotype can also immediately be used to selectively breed colonies that are more resistant [15].

Currently, quantitative trait loci (QTLs) associated with queen fertility [16,17], resistance to chalkbrood [18,19] and varroosis [20,21], and various types of behavior [22,23,24] have been identified. For example, hygiene behavior, which is a social behavior helps control various diseases of the offspring such as varroosis [25,26,27]. Varroosis is one of the most devastating diseases of the brood caused by the parasitic mite *Varroa destructor* [28,29,30]. Hygiene behavior has been shown to provide significant resistance against the *Varroa* mite [13,30]. QTL studies of *Varroa* resistance behavior in honey bees have identified over 20 suggestive QTLs in different genomic regions [14,20,21,22,31]. 

Like varroosis, nosemosis is also one of the most dangerous diseases [32,33,34], but the study of associations between molecular genetic markers and nosemosis is rare [35,36].

Nosemosis is a serious disease in adult honey bees caused by microsporidia, which are obligate intracellular eukaryotic parasites [37]. *Nosema* parasites multiply and develop within the host-cell cytoplasm causing extensive and even total destruction of the midgut epithelial layer [38,39].

In European honey bees, two species of microsporidia, *Nosema apis* and *Nosema ceranae*, have been described. *N. apis* Zander, 1909 [40] is an evolutionarily old parasite of the honey bee *A. mellifera*. The parasite causing type A nosemosis is moderately virulent, and bee colonies are able to resist disease under favorable environmental conditions [41,42,43]. *N. ceranae* Fries et al., 1996 [44], responsible for type C nosemosis, is a relatively new parasite for the *A. mellifera* [38,44,45,46]. This parasite was originally described in an Asian bee *Apis cerana* in the late 20th century [44]. Since 2006, it has been found in honey bee *A. mellifera* populations around the world [38,41,42,43,45,47,48,49,50,51,52,53,54]. Compared to *N. apis*, *N. ceranae* is considered a more virulent parasite, and in some countries, such as the Mediterranean countries, it is associated with the bee colony losses [32,55,56,57,58].

Using microsatellite markers, four QTLs associated with low spore load were revealed in a Danish selected *Nosema*-resistant honey bees line [35]. These Buckfast honey bee colonies have been selectively bred for the absence of *Nosema* over decades, resulting in a breeding line that is tolerant toward *Nosema* [59,60]. Unlike pure race breeding, the Buckfast breeding system mixes different stocks to establish a hybrid bee with desired characteristics. The Buckfast contains heritage from mainly *A. m. ligustica* and *A. m. mellifera* and from other subspecies. Since the Buckfast bee is a hybrid bee, the expression of its notable characteristics can vary greatly within the stock [61]. 

It is assumed that the honey bee subspecies (lines and colonies) differ in their resistance to disease, which may be determined by social immunity including hygienic and other types of behavior [13,15,30,62,63,64,65,66,67,68,69,70]. For example, in *A. mellifera*, hygienic and grooming behaviors are expressed more highly in Africanized honey bees than in European ones. Perhaps this explains the higher resistance of Africanized bees to *V. destructor* compared to European bees [71].

Although no significant effect of *N. ceranae* infections on hygienic behavior was detected [72], it is clear that natural resistance of honey bees to *Nosema* depends on many factors and the genetic variants of honey bees can play a relevant role. The purpose of this study was to identify associations between genetic variants of some microsatellite loci and *Nosema* infection/disease resistance in the dark forest bee *Apis mellifera mellifera*.

## 2. Materials and Methods

### 2.1. Bee Samples

In the present study, samples of a dark forest bee *Apis mellifera mellifera* from Siberian populations (longitude 81°29′−92°08′ E and latitude 50°44′−65°47′ N) were examined. The dark forest bee is a native bee that was introduced to Siberia about 230 years ago and has adapted well to the local climate and plant communities. In Siberia, the bee population is an artificial population; wintering of bees is controlled by people [73]. Honey bees were collected from 12 apiaries between the end of May 2016 and August of the same year. A total of 226 workers from twenty-eight bee colonies (from 8 to 10 bees from each bee colony) were examined.

For the diagnosis and detection of *Nosema* infection, the oldest honey bees (forager bees) were collected outside the entrance of hive, because they have the greatest infection and the highest proportion of infected bees [39]. Bee samples were stored in a freezer at −20 °C until further processing. 

### 2.2. Study Design

The present research has conducted in several stages. At the first stage of the study, the presence of *Nosema* spp. in honey bees was investigated using both light microscopy and polymerase chain reaction (PCR).

At the second stage of the study, the genetic diversity of honey bees with different degrees of *Nosema* infestation was examined using polymorphic microsatellite loci. Earlier, the genetic diversity of local dark forest bees (Siberian population) was studied using a complex of microsatellite loci and identified polymorphic microsatellite loci [74]. In this study, 23 polymorphic microsatellite loci were used to search for genetic markers of *Nosema* disease resistance in honey bees.

Finally, the associations of polymorphic variants of microsatellite loci studied with nosemosis were analyzed using the odds ratio method (OR).

### 2.3. Experimental Procedures

To carry out individual analysis of the bees, for each sample, the bee’s midgut was isolated and divided in two. One part of the midgut was used for light microscopy. For this, the midgut was ground in 0.5 mL of sterile, distilled water and the number of *Nosema* spores was counted using a Zeiss Axio Lab.1 light microscope.

DNA was extracted from another part of the midgut using a DNA purification kit, PureLink™ Mini (Invitrogen, Carlsbad, CA, USA) according to the manufacturer’s protocol. PCR was performed using a thermal MyCycler T100 (BioRad, Foster City, CA, USA).

For the diagnosis of nosemosis, duplex-PCR was used [42]. The primer sequences used to amplify the 321 bp fragment corresponding to the 16S ribosomal gene of *N. apis* were 321APIS-FOR 5′-GGGGGCATGTCTTTGACGTACTATGTA-3′ and 321APIS-REV 5′-GGGGGGCGTTTAAAATGTGAAACAACTATG-3′. The primer sequences utilized to amplify the 218 bp fragment corresponding to the 16S ribosomal gene of *N. ceranae* were 218MITOC-FOR 5′-CGGCGACGATGTGATATGAAAATATTAA-3′ and 218MITOC-REV 5′-CCCGGTCATTCTCAAACAAAAAACCG-3′ [42]. PCR was performed in a reaction volume of 20 µL containing 5–10 ng of template DNA, 1 × PCR buffer, 200 µM of each dNTP, 1.5 mM MgCl_2_, 0.2 µM of each primer and 1U Taq polymerase (Fermentas, Thermo Fisher Scientific, Chelmsford, MA, USA). The routine consisted of an initial denaturation step at 94 °C for 2 min, followed by 35 cycles of 94 °C for 30 s, 58 °C for 30 s and 72 °C for 1 min, and a final extension step at 72 °C for 5 min. PCR products were analyzed on 1.5% agarose gels. Gels were stained with ethidium bromide and visualized using UV illumination (Gel Doc XR+, BioRad, Foster City, CA, USA). For each PCR, positive control (reference *N. apis* and *N. ceranae DNA* extracts as template) was used. Negative control (ddH2O) was also included in each run of PCR amplification to detect possible contamination.

The variability of 23 polymorphic microsatellite loci (Ap066, K0457B, K1168, A007, A008, A028, A043, Ap049, Ap007, AC117, 6339, Ap068, Ap243, SV220, SV167, SV185, Ap226, H110, A024, AT139, A056, Ap249, and A113) was examined. When choosing loci, some factors such as a high level of polymorphism, maximum chromosomal coverage (the studied loci are located on 13 out of 16 honey bee chromosomes), and data from publications on DNA markers associated with bee resistance to diseases were considered. PCR was performed using specific fluorescence-labeled primers according to Solignac et al. [75]. The reactions were performed in 20 μL of a solution containing 5–10 ng DNA template, 0.4 µM of each primer, 60 µM of each dNTP, 1–2.5 mM MgCl_2_ and 1 × PCR buffer and 1U of Taq polymerase (Fermentas, Thermo Fisher Scientific, Chelmsford, MA, USA). After a denaturing step of 3 min at 94 °C, samples were processed through 35 cycles consisting of 30 s at 94 °C, 30 s at 55–60 °C and 30 s at 72 °C. The final elongation step was 10 min at 72 °C [75]. Amplification products were analyzed with ABI Prism 3730 Genetic Analyzer and GeneMapper Software (Applied Biosystems, Inc., Foster City, CA, USA) in the collective center Medical Genomics (Research Institute of Medical Genetics, Tomsk National Research Medical Center, Russian Academy of Sciences, Moscow, Russia). Two microliters of PCR products were mixed with GeneScan500-ROX size standards (Applied Biosystems, Inc.) and deionized formamide. Samples were run according to the manufacturer’s recommendations.

### 2.4. Estimation of the Nosemosis Level

According to PCR, most of the bees examined were coinfected with two *Nosema* species, *N. apis* and *N. ceranae*. In this regard, we considered the general infestation of honey bees with microsporidia, without division into *Nosema* species. A light microscope using 400× magnification was used for counting *Nosema* spores in macerated bee preparations.

Honey bees were divided into the following groups, uninfected (*Nosema*-negative) and *Nosema*-infected (*Nosema*-positive). Since our study did not require a high degree of precision, we used an arbitrary infection scale and divided the *Nosema*-positive bee group into two variants, *Nosema*-positive low and *Nosema*-positive high. *Nosema*-positive low bees were with a small amount of microsporidial spores on microscopic analysis (less than 100 spores in the field of view of the microscope). *Nosema*-positive high bees were with a significant number of microsporidial spores detected by microscopic analysis (more than 500 spores in the field of view of the microscope). An intermediate variant of the bee infection (100–500 *Nosema* spores in the field of view of the microscope) was not identified. In total, three groups of bees were formed: *Nosema*-negative, *Nosema*-positive low and *Nosema*-positive high.

### 2.5. Statistical Analysis

The genotypes obtained for each of the honey bee were used to estimate population parameters. The allelic and genotypic observed frequencies by Hardy–Weinberg equilibrium (HWE), the number of alleles, observed (Ho) and expected (He) heterozygosity were estimated employing the GENEPOP v.4.1 package [76]. To assess genetic diversity, for each infectious category, the observed and expected heterozygosity for each locus were compared using a Student’s test. Comparison of allele and genotype frequencies between bee samples that differed in *Nosema* infestation was performed using the chi-square test. In the case of a small number of one of the comparison classes, the chi-square test with Yates’ correction was used.

To assess the association of polymorphic variants of the microsatellite loci studied with *Nosema* infection in bees, the odds ratio (OR) and 95% confidence interval (95% CI) corresponding to the *p*-value was calculated [77]. The association of the genetic marker with the tested trait was determined by the OR value when the differences in the allele frequency between the compared groups reached the level of statistical significance, *p* < 0.05. If the OR > 1, the assumption about the association of the analyzed genetic variant (allele/genotype) with the studied pathology is considered (increased chance of developing disease). When the OR < 1, a protective role of the corresponding genetic variant (allele/genotype) is assumed.

## 3. Results

### 3.1. Genetic Diversity of the A. m. mellifera Honey Bees in Siberia on the Microsatellite Loci

Honey bees with different degrees of *Nosema* infestation were genotyped using 23 microsatellite markers. When comparing the distribution of allele frequencies between three groups of the *A. m. mellifera* bees (*Nosema*-negative, *Nosema*-positive low and *Nosema*-positive high), several loci (AC117, A113, Ap243, A024, A007, Ap049 and SV185) were identified that are promising for further analysis. The parameters of genetic diversity of these loci, such as the frequencies of alleles and genotypes, expected heterozygosity and observed heterozygosity, are presented in Table 1.

All microsatellite loci studied were polymorphic: the minimum number of alleles was found for locus A007 (3 alleles), and the maximum number of alleles was for loci A113 and Ap243 (8 alleles); the average number of alleles per locus was 5 (Table 1). 

Some studied loci differed in the variability in honey bees of different infectious categories (uninfected and *Nosema*-infected bees). For example, for the Ap243 locus, the frequency of the predominant allele “256” differs in bees of two *Nosema*-positive groups, *Nosema*-positive low and *Nosema*-positive high bees (t = 2.30; *p* < 0.05). Interestingly, the frequency of the other allele (“263”) of the Ap243 locus was also statistically significantly different between uninfected (*Nosema*-negative) and significantly *Nosema*-infected (*Nosema*-positive high) bees (t = 2.61; *p* < 0.05) and between two *Nosema*-positive groups (t = 5.10; *p* < 0.001). In addition, the frequency of alleles “177” and “181” of the AC117 locus differs between *Nosema*-negative and *Nosema*-positive high bees (t = 2.46; *p* < 0.05 and t = 3.09; *p* < 0.05, respectively), and between bees of two *Nosema*-positive groups (t = 2.27; *p* < 0.05 and t = 5.15; *p* < 0.01, respectively).

An assessment of the heterozygosity of most of the studied loci (except for loci A007 and SV185 in *Nosema*-positive high bees) revealed lower values of the observed heterozygosity (Ho) compared with the expected heterozygosity (He) (Table 1). A statistically significant level of differences between the values of the observed and expected heterozygosity is shown for the following loci: A113 (t = 3.82, *p* ˂ 0.001 and t = 3.16, *p* ˂ 0.05), Ap243 (t = 4.35, *p* ˂ 0.001 and t = 2.09, *p* ˂ 0.05) in *Nosema*-positive low and high bees, respectively; SV185 (t = 2.77, *p* ˂ 0.05) in *Nosema*-positive low bees; AC117 (t > 3.69, *p* ˂ 0.001) in all infection categories of bees.

Thus, the analysis of the variability of 23 microsatellite loci in the *A. m. mellifera* honey bees allowed us to identify the most promising loci for searching for associations of DNA markers with the *Nosema* disease.

### 3.2. Comparative Characteristics of the Genetic Diversity of A. m. mellifera Bees from Different Infectious Categories

The heterogeneity of bee groups differing in the degree of *Nosema* infection was evaluated. To do this, statistically significant differences between the bee groups in allele frequencies of microsatellite loci were determined.

For some microsatellite loci (AC117, A113, Ap243 and Ap049), differences in the distribution of allele frequencies (in total) between infectious categories of bees are shown. Mainly, differences were found between infected bees (*Nosema*-positive low and *Nosema*-positive high bees): locus AC117 (χ^2^ = 44.61, df = 7, *p* ˂ 0.01); locus A113 (χ^2^ = 12.76, df = 5, *p* ˂ 0.05); locus Ap243 (χ^2^ = 19.77, df = 7, *p* ˂ 0.01) and locus Ap049 (χ^2^ = 14.70, df = 7, *p* ˂ 0.05). In addition, for the AC117 locus, differences were revealed between uninfected and *Nosema*-positive high bees (χ^2^ = 19.84, df = 7, *p* ˂ 0.01). No statistically significant differences were found between the groups of uninfected bees and *Nosema*-positive low bees.

For the locus AC117, the alleles “177”, “181” and “185” make the largest contribution. The “177” allele is most often found in uninfected bees than *Nosema*-positive high bees (χ^2^ = 9.59, df = 1, *p* ˂ 0.01). On the contrary, the “181” allele is more typical for *Nosema*-positive high bees than for uninfected (χ^2^ = 8.66, df = 1, *p* ˂ 0.01) and *Nosema*-positive low (χ^2^ = 26.56, df = 1, *p* ˂ 0.01) bees. For the “185” allele, differences were found between the two groups of infected bees (χ^2^ = 14.17, df = 1, *p* ˂ 0.01).

For the locus A113, the allele “218” defines the differences between *Nosema*-negative and *Nosema*-positive high bees (χ^2^ = 6.81, df = 1, *p* ˂ 0.01). In addition, significant differences were also found between *Nosema* infected (low and high) bees on the frequency of the alleles “218” (χ^2^ = 6.12, df = 1, *p* ˂ 0.05) and “212” (χ^2^ = 6.60, df = 1, *p* ˂ 0.01).

For the locus Ap243, the frequencies of two alleles “256” and “263” were significantly different in the groups of infected bees (χ^2^ = 5.80, df = 1, *p* ˂ 0.05 and χ^2^ = 12.45, df = 1, *p* ˂ 0.01, respectively). Allele “256” prevailed in *Nosema* infected high bees, while allele “263” was more common in *Nosema* infected low bees (Table 1). The allele “263” also determined differences between groups of uninfected and *Nosema*-positive high bees (χ^2^ = 6.98, df = 1, *p* ˂ 0.01).

For the locus Ap049, alleles “127” and “130” determine the differences between groups of infected bees (χ^2^ = 4.00, df = 1, *p* ˂ 0.05 and χ^2^ = 5.12, df = 1, *p* ˂ 0.05, respectively), while allele “120” was between uninfected and *Nosema*-positive high bees (χ^2^ = 4.69, df = 1, *p* ˂ 0.05).

Despite the fact that no significant differences were found in the distribution of all alleles (in total) of loci A007, A024 and SV185, there existed differences for some alleles of these loci. For example, allele “269” of the SV185 locus determined the differences between groups of *Nosema*-uninfected and *Nosema*-positive high bees (χ^2^ = 5.65, df = 1, *p* ˂ 0.05).

### 3.3. Assessment of Associations of Genetic Markers with Nosema Infection/Resistance in the Dark Forest Bee A. m. mellifera

In order to search for alleles, possibly associated with *Nosema* disease in the dark forest bees (*A. m. mellifera*), the odds ratio, OR, was calculated (Table 2).

For loci AC117, A113, Ap243, A024, A007, Ap049 and SV185, statistically significant differences in allele and/or genotype frequencies between the compared groups (infection categories) were shown. However, according to OR calculations, only some genetic variants of these loci showed associations with *Nosema* infestation. Based on the calculated OR, it can be concluded that alleles “177” of the AC117 locus, “263” of the Ap243 locus and “269” of the SV185 locus have protective properties (that is, they reduce the risk of *Nosema* infection).

It is worth pointing out that the frequency of homo- and heterozygous genotypes with an allele “177” of the AC117 locus decreased in the sequence: uninfected bees (25.9% of *Nosema*-negative individuals) were weakly infected (14.1% of *Nosema*-positive low bees) and significantly infected (4.9% of *Nosema*-positive high individuals) (Table 1). Between the extreme compared groups of bees (*Nosema*-negative and *Nosema*-positive high) differences in the frequency of individuals with genotypes having this allele reach the level of statistical significance (χ^2^ = 16.61, df = 2, *p* ˂ 0.01). For the Ap243 locus, the frequency of genotypes with the “263” allele in *Nosema*-positive high bees (11.1%) differed significantly from both uninfected bees (43.3%; χ^2^ = 13.45, df = 6, *p* ˂ 0.05) and *Nosema*-positive low individuals (43.5%; χ^2^ = 18.86, df = 7, *p* ˂ 0.01) (Table 1). For the SV185 locus, there were no differences between the compared groups at the genotype level (*p* > 0.05). In addition, the results obtained suggest that the “218” allele of the A113 locus is associated with an increased risk of nosemosis in *A. m. mellifera* bees (no differences were recorded at the genotype level).

## 4. Discussion

The genetic diversity of the *A. m. mellifera* bees, differing in the degree of *Nosema* infection, was investigated, and a search for associations between genetic variants of microsatellite loci and *Nosema* disease was carried out. The present results show that some alleles of microsatellite loci are associated with nosemosis in the *A. m. mellifera* bees living in the Siberian region. For example, alleles “177” of the AC117 locus, “263” of the Ap243 locus and “269” of the SV185 locus reduce the risk of *Nosema* infection.

This study found associations of genetic markers with *Nosema* infection/disease resistance in honey bees and was an exploratory study. Therefore, it is necessary to understand whether the results obtained were random or reflected some general biological regularities. Possible solutions to this issue are expanding the bee samples and/or analyzing different bee subspecies and studying them in terms of the importance of microsatellite loci in determining resistance to nosemosis. 

Previously, the associations of some microsatellite loci in the *Apis mellifera carpathica* bees were analyzed by us together with T. Kireeva (Tomsk State University, our unpublished data) [78]. Interestingly, for the Ap243 locus, statistically significant differences were also shown between uninfected and *Nosema*-infected *A. m. carpathica* bees (χ^2^ = 22.93, df = 7, *p* ˂ 0.01). Alleles “253” and “256” determine the differences between groups of uninfected and *Nosema*-infected bees (χ^2^ = 9.69, df = 1, *p* ˂ 0.01 and χ^2^ = 7.03, df = 1, *p* ˂ 0.01, respectively). Based on the calculated OR, it can be concluded that allele “256” of the Ap243 locus has protective properties (OR = 0.29, 95% CI—0.10–0.84, χ^2^/*p*—6.87/0.0088, χ^2^-Yeats/*p*—5.57/0.0182), while allele “253”, on the contrary, increased the risk of *Nosema* infection (OR = 3.57, 95% CI—1.53–8.40, χ^2^/*p*—11.10/0.00086, χ^2^-Yeats/*p*—9.77/0.0018).

Thus, among the investigated microsatellite loci, the Ap243 locus located on chromosome 1 (group 1.1) was shared for two bee subspecies (*A. m. mellifera* and *A. m. carpathica*) and is probably associated with the incidence/resistance to nosemosis. However, in these subspecies, different alleles associated with *Nosema* disease were identified. For *A. m. mellifera* bees, allele “263” probably reduced the risk of infection with nosemosis (protective role), and statistically significant differences were also found at the genotype level in bees of different groups (*Nosema*-positive high bees differed from uninfected and *Nosema*-positive low bees). In *A. m. carpathica* bees, statistically significant differences were found for two alleles (“256” and “253”) between uninfected bees and *Nosema*-infected individuals, and the allele “256” is likely to have a protective meaning, while the allele “253”, on the contrary, is associated with a disease.

In addition, the A024 locus is also of interest for studying its association with nosemosis. Despite the fact that in the *A. m. mellifera* bees, no allele of the A024 locus showed association with *Nosema* disease (Table 2), in the *A. m. carpathica* bees, the allele “90” probably determines the resistance to nosemosis (OR = 0.09, 95% CI—0.04–0.023, χ^2^/*p*—39.94/0.0000000, χ^2^-Yeats/*p*—37.93/0.0000000).

It should be noted that for two bee subspecies, various microsatellite loci and alleles associated with the *Nosema* incidence/resistance have been identified, which may be determined by the different resistance of bee subspecies to nosemosis and/or by different habitats of bees (geographic, natural, climatic and nutritional conditions) [58,79,80,81,82]. Perhaps, in addition to the subspecies-specific features to *Nosema* resistance, the revealed differences can be determined by the structure of the chromosomal region where the QTL is located. For example, not this locus itself, but another, which is in linkage disequilibrium with it, may be involved in the determination of resistance to nosemosis, i.e., different alleles are in the same linkage group with a favorable variant in different bee subspecies.

The few hygienic behavior-related studies focused on finding quantitative trait loci controlling *Varroa* resistance hygienic behavior of honey bees have also identified different chromosomal regions related to hygienic behavior and reduced mite reproduction [14,20,21,22,31]. QTL studies of *Varroa* resistance behavior have identified over 20 suggestive chromosome regions associated with linkage groups 2, 3, 4, 5, 6, 7, 9, 10, 13, 15, 16 and 22. For example, using RAPD markers, Lapidge et al. (2002) found seven suggestive QTLs controlling hygienic behavior of honey bees [22]. Using microsatellite loci, Oxley et al. (2010) identified three significant and three suggestive QTLs that influence a honey bee worker’s propensity to engage in hygienic behavior [31] whereas Behrens et al. (2011) identified three QTLs controlling reduced mite reproduction in the Swiss *Varroa* mite tolerant honey bee lineage [20]. In the QTL study presented by Spötter et al., six SNPs showed significant genome-wide associations with hygienic behavior against *Varroa* at the genotype level [14]. 

It is worth pointing out that the presented studies did not identify the same (common) QTLs associated with hygienic behavior of honey bees. For example, Oxley et al. (2010) and Behrens et al. (2011) identified two QTLs for the trait in different regions of chromosome 9 [20,31]. Additionally, Tsuruda et al. (2012) also reported one major QTL on chromosome 9, but in a different region, for hygienic behavior against *Varroa* using a small-scale SNP-Chip [21]. Thus, the identified genomic regions related to hygienic behavior are different, which can be explained by different bee materials (freeze-killed brood, brood and worker bees), different research methods used and DNA markers analyzed (RAPD markers, microsatellite loci or SNP), different genetic maps (map based on RAPD or microsatellite markers). Finally, the use of different bee subspecies can significantly affect the research outcome. For example, in QTL studies presented by Oxley et al. (2010) and Spötter et al. (2016), QTLs were identified in the same chromosomes (chromosomes 2 and 5), but at different ends of the chromosome [14,31]. It is assumed that different bee material used in these studies contributed to the differences in the genomic regions identified [14]. On the one hand, a freeze-killed brood instead of brood that was artificially infested with *Varroa* was used in Oxley et al.’s research [31]. On the other hand, two different bee subspecies (*Apis mellifera ligustica* and *Apis mellifera carnica*) have been investigated [14].

A comparison between the QTLs involved in *N. ceranae* infection tolerance according to Huang et al.’s study [35] and our own trait-associated regions found no agreement. So, four QTLs located on chromosomes 3, 10, 6 and 14 were significantly associated with low *Nosema* spore load, explaining 20.4% of total spore load variance in the selected *Nosema*-resistant Danish honey bee strain. The significant QTL on chromosome 14 explains 7.7% of the total variance and may be responsible for the resistance to nosemosis in the selected Danish honey bee. A candidate gene *Aubergine* (*Aub*) within this QTL region was significantly more overexpressed in drones with a low spore load than in those with a high spore load [35].

From the data presented, in the dark forest bee, microsatellite loci Ap243 (chromosome 1), SV185 (chromosome 5) and AC117 (chromosome 12) are associated with resistance to nosemosis. It is interesting to note that in the chromosome region 1.1, the microsatellite locus Ap243 and the honey bee microRNA (ame-miR-2b) are located. As shown, host microRNAs respond to infection by the parasite *N. ceranae* [36]. In honey bees, 17 miRNAs were differentially expressed during *N. ceranae* infection, which may target over 400 predicted genes for ion binding, signaling, the nucleus, transmembrane transport and DNA binding. MicroRNA ame-miR-2b is particularly interesting because 11 out of 27 enzymes were significantly correlated with its expression level [36]. In addition, in the same region on chromosome 1, a suggestive QTL associated with the performance of *Varroa* sensitive hygiene was identified [21]. This locus contains more than candidate 30 genes [21], including puromycin-sensitive aminopeptidase involved in proteolytic events essential for cell growth and viability [83], selenoprotein F located in the endoplasmic reticulum and regulated by cell stress conditions [84], transcription and splicing factors and other genes. Since Microsporidian *Nosema* species are intracellular parasite [37], of particular interest is the cell wall integrity and stress response component 1. In *Schizosaccharomyces pombe*, the homologous gene *wsc1* is responsible for such biological processes as a cell surface receptor signaling pathway, intracellular signal transduction and regulation of cell wall organization or biogenesis [85].

Despite the fact that numerous QTLs associated with the disease resistance of honey bee have been identified, it is assumed that the variations in this trait is controlled by a small number of loci. For example, a strong genetic component is involved in the control of hygienic behavior, although it may also be influenced by environmental factors to some extent [14]. Therefore, the identification of the gene variants that are responsible for disease resistance in honey bees, and then breeding resistant bee colonies is one promising approach in the fight against bee disease.

## 5. Conclusions

The present study examined the associations between several variants of microsatellite loci and *Nosema* disease. In the dark forest bee, genetic markers promising for the assessment of nosemosis resistance, such as the allele “177” of the locus AC117, the allele “263” of the locus Ap243, the allele “269” of the locus SV185 have been identified. At the same time, some issues, for example, differences in the spectrum of loci and/or alleles that determine resistance to nosemosis in different bee subspecies/breeds, remained unresolved. In this regard, additional research both for the same bee subspecies/breeds bred in other regions, and for other bee subspecies/breeds is needed. However, already at this stage, these markers can be used to predict the risk of developing nosemosis, but with the obligatory consideration of the specificity of diagnostic markers for different bee subspecies/breeds.

## Figures and Tables

**Table 1:** Genotype and allele frequencies and heterozygosity at 7 microsatellite loci in the dark forest bees with varying degrees of *Nosema* infection.

Locus	Genotype	Allele	Infection Categories of Honey Bees					
			*Nosema*-Negative		*Nosema*-Positive Low		*Nosema*-Positive High	
			Genotype	Allele	Genotype	Allele	Genotype	Allele
AC117	173–173	173	0.037	0.074 ± 0.036	0.024	0.079 ± 0.017		0.057 ± 0.021
	173–181	177	0.074	0.148 ± 0.048	0.110	0.075 ± 0.017	0.115	0.025 ± 0.014
	177–177	181	0.037	0.296 ± 0.062	0.008	0.260 ± 0.028		0.533 ± 0.045
	177–181	185		0.482 ± 0.068	0.039	0.587 ± 0.031		0.385 ± 0.044
	177–185		0.222		0.094		0.049	
	181–181		0.259		0.142		0.475	
	181–185				0.087			
	185–185		0.370		0.496		0.361	
	Ho/He		0.296 ± 0.088 **/0.653 ± 0.040		0.331 ± 0.042 **/0.577 ± 0.025		0.164 ± 0.047 **/0.564 ± 0.025	
	N		27		127		61	
A113	210–218	210			0.016	0.008 ± 0.006		
	212–212	212		0.107 ± 0.041	0.063	0.152 ± 0.023	0.016	0.063 ± 0.021
	212–214	214	0.036	0.018 ± 0.018	0.008	0.008 ± 0.006		
	212–218	218	0.143	0.518 ± 0.067	0.125	0.598 ± 0.031	0.094	0.719 ± 0.040
	212–220	220	0.036	0.339 ± 0.063	0.023	0.207 ± 0.025		0.219 ± 0.037
	212–222	222			0.008	0.008 ± 0.006		
	212–226	226			0.016	0.020 ± 0.009		
	214–226	228		0.018 ± 0.018	0.008			
	218–218		0.286		0.438		0.609	
	218–220		0.321		0.172		0.125	
	218–222				0.008			
	220–220		0.143		0.109		0.156	
	220–228		0.036					
	226–226				0.008			
	Ho/He		0.571 ± 0.094/0.605 ± 0.041		0.383 ± 0.043 **/0.577 ± 0.027		0.219 ± 0.052 */0.432 ± 0.043	
	N		28		128		64	
Ap243	253–260	253	0.043	0.022 ± 0.022			0.074	0.037 ± 0.026
	256–256	256	0.305	0.413 ± 0.073	0.284	0.419 ± 0.035	0.519	0.593 ± 0.067
	256–263	260	0.218	0.109 ± 0.046	0.233	0.111 ± 0.022	0.074	0.167 ± 0.051
	256–266	263		0.239 ± 0.063		0.283 ± 0.032	0.074	0.056 ± 0.031
	256–269	266		0.022 ± 0.022	0.030	0.010 ± 0.007		0.037 ± 0.026
	256–272	269		0.109 ± 0.046	0.010	0.096 ± 0.021		0.037 ± 0.026
	260–260	272	0.043	0.022 ± 0.022	0.081	0.035 ± 0.013	0.111	0.056 ± 0.031
	260–263	275	0.043	0.065 ± 0.036	0.010	0.046 ± 0.015		0.019 ± 0.018
	260–266		0.043		0.020			
	260–269				0.030		0.037	
	263–263		0.043		0.132			
	263–269		0.043		0.030			
	263–272		0.087		0.010		0.037	
	263–275				0.020			
	269–269		0.043		0.020			
	269–272				0.020		0.037	
	269–275		0.087		0.040			
	272–275				0.030		0.037	
	Ho/He		0.565 ± 0.103/0.743 ± 0.043		0.485 ± 0.050 **/0.719 ± 0.020		0.370 ± 0.093 */0.610 ± 0.067	
	N		23		99		27	
A024	92–92	92	0.500	0.712 ± 0.063	0.446	0.654 ± 0.030	0.313	0.578 ± 0.044
	92–100	96	0.231		0.177	0.008 ± 0.005	0.219	
	92–106	100	0.192	0.154 ± 0.050	0.238	0.181 ± 0.024	0.313	0.266 ± 0.039
	96–96	102			0.008	0.008 ± 0.005		
	100–100	106	0.038	0.135 ± 0.047	0.077	0.150 ± 0.022	0.156	0.156 ± 0.032
	100–102				0.015			
	100–106				0.015			
	106–106		0.038		0.023			
	Ho/He		0.423 ± 0.097/0.452 ± 0.070		0.446 ± 0.044/0.517 ± 0.029		0.531 ± 0.062/0.571 ± 0.032	
	N		26		130		64	
A007	104–108	104	0.185	0.093 ± 0.039	0.224	0.121 ± 0.021	0.364	0.182 ± 0.041
	104–113	108		0.815 ± 0.053	0.017	0.797 ± 0.026		0.818 ± 0.041
	108–108	113	0.704	0.093 ± 0.039	0.655	0.082 ± 0.018	0.636	
	108–113		0.037		0.060			
	113–113		0.074		0.043			
	Ho/He		0.222 ± 0.080/0.319 ± 0.075		0.302 ± 0.043/0.343 ± 0.036		0.364 ± 0.073/0.298 ± 0.052	
	N		27		116		44	
Ap049	120–120	120	0.036	0.161 ± 0.049	0.017	0.121 ± 0.021		0.057 ± 0.022
	120–127	127	0.250	0.714 ± 0.060	0.200	0.646 ± 0.031	0.113	0.745 ± 0.042
	120–130	130		0.054 ± 0.030	0.008	0.175 ± 0.025		0.085 ± 0.027
	127–127	139	0.536	0.071 ± 0.034	0.425	0.046 ± 0.014	0.585	0.085 ± 0.027
	127–130	152	0.036		0.192	0.013 ± 0.007	0.094	0.028 ± 0.016
	127–139		0.071		0.050		0.113	
	130–130		0.036		0.067		0.019	
	130–139						0.019	
	130–152				0.017		0.019	
	139–139		0.036		0.017		0.019	
	139–152				0.008			
	152–152						0.019	
	Ho/He		0.357 ± 0.091/0.456 ± 0.071		0.475 ± 0.046/0.535 ± 0.032		0.359 ± 0.066/0.426 ± 0.057	
	N		28		120		53	
SV185	253–253	253			0.009	0.022 ± 0.010		
	253–272	263		0.241 ± 0.058	0.027	0.313 ± 0.031		0.385 ± 0.050
	263–263	266	0.037	0.093 ± 0.039	0.134	0.094 ± 0.020	0.146	0.146 ± 0.036
	263–266	269		0.667 ± 0.064	0.045	0.549 ± 0.033	0.125	0.469 ± 0.051
	263–269	272	0.407		0.304	0.023 ± 0.010	0.354	
	263–272				0.009			
	266–266		0.074		0.045			
	266–269		0.037		0.054		0.167	
	269–269		0.444		0.366		0.208	
	269–272				0.009			
	Ho/He		0.444 ± 0.096/0.489 ± 0.061		0.446 ± 0.047 */0.591 ± 0.023		0.646 ± 0.069/0.611 ± 0.023	
	N		27		112		48	

N—the number of bee samples analyzed within each infection category; Ho—observed heterozygosity; He—expected heterozygosity according to Hardy–Weinberg equilibrium. In the table, the values of allele frequencies and parameters of heterozygosity with a standard error are given. Statistically significant differences in the observed heterozygosity from the expected heterozygosity are marked with (*) (* *p* ˂ 0.05, ** *p* ˂ 0.001).

**Table 2:** Comparative analysis of the frequency of potentially significant genetic variants in the formation of nosemosis resistance in the dark forest bee *A. m. mellifera*.

Locus	Compared Alleles/Genotypes	Parameters	Compared Honey Bee Groups		
			*Nosema*-Negative–*Nosema*-Positive Low	*Nosema*-Negative–*Nosema*-Positive High	*Nosema*-Positive Low–*Nosema*-Positive High
AC117	Allele 177 vs. others	OR	0.46	**0.16**	0.35
		95% CI	0.18–1.24	**0.04**–**0.58**	0.11–1.13
		χ^2^/*p*	2.15/0.17	**7.76**/**0.005**	2.92/0.09
	Homo- and heterozygous genotypes with an allele 177 vs. others	OR	0.47	**0.16**	0.35
		95% CI	0.16–1.43	**0.04**–**0.64**	0.11–1.16
		χ^2^/*p*	1.48/0.22	**6.25/0.01**	2.69/0.10
A113	Allele 218 vs. others	OR	1.38	**2.38**	**1.72**
		95% CI	0.74–2.57	**1.18**–**4.80**	**1.04**–**1.39**
		χ^2^/*p*	0.90/0.34	**6.12**/**0.01**	**4.91**/**0.03**
Ap243	Allele 263 vs. others	OR	1.25	**0.21**	**0.17**
		95% CI	0.27–2.83	**0.06**–**0.75**	**0.06**–**0.53**
		χ^2^/*p*	0.17/0.68	**5.51**/**0.02**	**10.99**/**0.0009**
	Homo- and heterozygous genotypes with an allele 263 vs. others	OR	1.00	**0.18**	**0.19**
		95% CI	0.37–2.74	**0.05**–**0.73**	**0.06**–**0.51**
		χ^2^/*p*	0.05/0.82	**5.19**/**0.02**	**8.22**/**0.004**
A024	Allele 92 vs. others	OR	0.77	0.56	0.73
		95% CI	0.38–1.53	0.26–1.17	0.46–1.15
		χ^2^/*p*	0.41/0.52	2.25/0.13	1.80/0.18
	Allele 100 vs. others	OR	1.21	1.99	1.64
		95% CI	0.51–3.00	0.80–5.10	0.96–2.16
		χ^2^/*p*	0.07/0.79	2.00/0.16	3.24/0.07
A007	Allele 104 vs. others	OR	1.35	2.18	1.62
		95% CI	0.46–4.19	0.69–7.32	0.78–3.32
		χ^2^/*p*	0.12/0.73	1.47/0.23	1.53/0.22
Ap049	Allele 120 vs. others	OR	0.72	0.31	0.44
		95% CI	0.30–1.76	0.09–1.04	0.16–1.15
		χ^2^/*p*	0.34/0.56	3.57/0.06	2.67/0.10
SV185	Allele 269 vs. others	OR	0.61	**0.44**	0.72
		95% CI	0.31–1.18	**0.21**–**0.93**	0.44–1.20
		χ^2^/*p*	2.00/0.16	**4.68**/**0.03**	1.43/0.23

OR—odds ratio, 95% CI—limits of the 95% confidence interval, χ^2^/*p*—χ^2^ test and its level of significance, df = 1. Alleles for which the level of statistical significance on OR has been reached are indicated in bold.

## Data Availability

Data is contained within the article or Supplementary Material. The data presented in this study are available in [Ostroverkhova, N.V.; Kucher, A.N.; Konusova, O.L.; Kireeva, T.N.; Rosseykina, S.A.; Yartsev, V.V.; Pogorelov, Y.L. Genetic diversity of honey bee *Apis mellifera* in Siberia. In *Phylogenetics of Bees*; Ilyasov, R.A., Kwon, H.W., Eds.; CRC Press: Boca Raton, FL, USA, 2020; pp. 97–126 and Ostroverkhova, N.V.; Kucher, A.N.; Konusova, O.L.; Gushchina, E.S.; Yartsev, V.V.; Pogorelov, Y.L. Dark-Colored Forest Bee *Apis mellifera* in Siberia, Russia: Current State and Conservation of Populations. In *Selected Studies in Biodiversity*; IntechOpen: London, UK, 2018; pp. 157–180].
